# Sympatric Spawning but Allopatric Distribution of *Anguilla japonica* and *Anguilla marmorata* **:** Temperature- and Oceanic Current-Dependent Sieving

**DOI:** 10.1371/journal.pone.0037484

**Published:** 2012-06-04

**Authors:** Yu-San Han, Apolinario V. Yambot, Heng Zhang, Chia-Ling Hung

**Affiliations:** 1 Institute of Fisheries Science, College of Life Science, National Taiwan University, Taipei, Taiwan; 2 College of Fisheries–Freshwater Aquaculture Center, Central Luzon State University, Science City of Munoz, Philippines; 3 East China Sea Fisheries Research Institute, Chinese Academy of Fishery Sciences, Shanghai, China; Institut Pluridisciplinaire Hubert Curien, France

## Abstract

*Anguilla japonica* and *Anguilla marmorata* share overlapping spawning sites, similar drifting routes, and comparable larval durations. However, they exhibit allopatric geographical distributions in East Asia. To clarify this ecological discrepancy, glass eels from estuaries in Taiwan, the Philippines, Indonesia, and China were collected monthly, and the survival rate of *A. marmorata* under varying water salinities and temperatures was examined. The composition ratio of these 2 eel species showed a significant latitude cline, matching the 24°C sea surface temperature isotherm in winter. Both species had opposing temperature preferences for recruitment. *A. marmorata* prefer high water temperatures and die at low water temperatures. In contrast, *A. japonica* can endure low water temperatures, but their recruitment is inhibited by high water temperatures. Thus, *A. japonica* glass eels, which mainly spawn in summer, are preferably recruited to Taiwan, China, Korea, and Japan by the Kuroshio and its branch waters in winter. Meanwhile, *A. marmorata* glass eels, which spawn throughout the year, are mostly screened out in East Asia in areas with low-temperature coastal waters in winter. During summer, the strong northward currents from the South China Sea and Changjiang River discharge markedly block the Kuroshio invasion and thus restrict the approach of *A. marmorata* glass eels to the coasts of China and Korea. The differences in the preferences of the recruitment temperature for glass eels combined with the availability of oceanic currents shape the real geographic distribution of *Anguilla japonica* and *Anguilla marmorata*, making them “temperate” and “tropical” eels, respectively.

## Introduction

Freshwater eels (genus *Anguilla*) are catadromous fish and have a complex life history [1]. All anguillids spawn in the tropical/subtropical ocean and have leptocephalus larvae that disperse from their oceanic spawning area to continental habitats via ocean currents where they metamorphose into glass eels [2]–[5]. After growing for years in rivers and estuaries, they return to their birthplace to spawn and subsequently die. Nineteen species and subspecies of genus *Anguilla* are reported worldwide; 6 of them are temperate eels that mostly have well-defined spawning and recruitment seasons [1], [3], long larval durations [3], and panmictic populations [6]–[8]. In contrast, tropical eels usually have year-round recruitment because of protracted spawning seasons [3], [9]–[11] and shorter larval durations than those of temperate eels [3], [9], and may have multiple populations/spawning areas such as those of *Anguilla marmorata* [12]–[14].

In general, temperate eels are distributed mainly in subtropical/temperate areas, whereas tropical eels are distributed mainly in tropical/subtropical regions [1]. Despite the different biogeographical distributions of these 2 types of eels, some of them utilize the same oceanic currents for larval transportation. For example, *A. japonica*, *A. marmorata*, and *A. bicolor pacifica* are transported by the North Equatorial Current (NEC) [3], [11], [15]. Meanwhile, *A. bicolor bicolor*, *A. nebulosa labiata*, *A. mossambica*, and *A. marmorata* in East Africa are transported by the South Equatorial Current [4]. Moreover, *A. australis* and *A. reinhardtii* use the South Equatorial Current for larval transportation [2], [16]. However, why eel species utilizing the same oceanic currents for transportation have different biogeographical distributions is poorly understood. Some differences in life history strategies between temperate and tropical eels may be responsible for their adaptation to different climate patterns and geographical environments.

**Figure 1:**
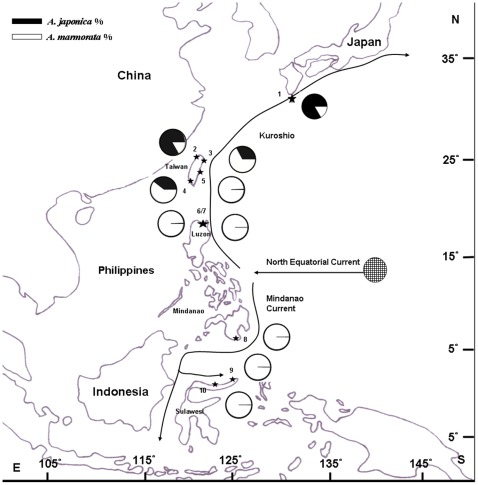
Map showing the relative abundances of *A. japonica* and *A. marmorata* in East Asia. The sampling locations of the glass eels are indicated by asterisks. No. 1: Yakushima Is., n = 2050 (Yamamoto et al., 2001); No. 2: Danshui R., n = 2547; No. 3: Yilan R., n = 3279; No. 4: Tungkang R., n = 673; No. 5: Siouguluan R., n = 7170; No. 6: Cagayan R., n = 1974 (Tabeta et al., 1976); No. 7: Cagayan area., n = 2075; No. 8: Buayan R., n = 552; No. 9: Manado, n = 350; No. 10: Poigar R., n = 4997 (Arai et al., 1999).

**Table 1:** Primers used for eel species identification.

Primer name	Sequence (5′-3′)
Cytochrome *b*, universal forward	5′-GATGCCCTAGTGGATCTACC-3′
Cytochrome *b*, universal reverse	5′-TATGGGTGTTCTACTGGTAT-3′
Cytochrome *b*, *A. marmorata*	5′-GTCAACTGAAAAGCCTCCTCAA-3′
Cytochrome *b*, *A. celebesensis*	5′-CAGATTCATTGGACTAGGG-3′
Cytochrome *b*, *A. luzonensis*	5′-ATCAACTGAGAAGCCCCCTCAG-3′

The tropical giant mottled eel, *A. marmorata*, has the widest geographic distribution among *Anguilla* species; it is distributed longitudinally from the east coast of Africa to the central South Pacific and latitudinally from Japan to Southern Africa [11], [17]. Genetic and morphological studies show that this species consists of several distinct spawning populations [13], [14], [18]. One spawning population known as the North Pacific population ranges from Northern Indonesia to Southern Japan [11], [13], [14]. According to surveys of larvae [14] and mature silver eels [19] collected from the NEC region, the spawning area of *A. marmorata* is around 12–17° N, 131–143°E [11], [15], which overlaps with that of the Japanese eel *A. japonica* (12–17° N, 137–143°E) [20], [21]. Newly hatched *A. japonica* and *A. marmorata* larvae in the NEC drift west from their spawning area. When the NEC arrives at the east coast of the Philippines, it divides into northbound (Kuroshio) and southbound flows (Mindanao Current). The leptocephali rely on these oceanic currents for transportation before metamorphosing into glass eels [5], [11], [21]. *A. japonica* and *A. marmorata* metamorphose at similar ages: at around 3–5 months of age [3], [22]–[24]. The overlapping spawning areas, transportation routes, and leptocephalus durations between *A. marmorata* and *A. japonica* suggest that both should be dispersed in similar geographic areas. However, the real distribution ranges of these 2 eel species are distinct. *A. japonica* glass eels are found mainly in Taiwan, China, Korea, and Japan [5], [11], [24], [25]. Meanwhile, *A. marmorata* glass eels are abundant in the Philippines and Indonesia [9]–[11], [26]. The sympatric spawning but allopatric distribution between *A. japonica* and *A. marmorata* is an interesting ecological phenomenon.

**Table 2:** Collection date, location, and numbers of *A. marmorata* and *A. japonica* specimens.

Site	China		Taiwan								Philippines				Indonesia	
	SH		DS		YL		DG		SL		CA		BY		MD	
Date	Aj	Am	Aj	Am	Aj	Am	Aj	Am	Aj	Am	Aj	Am	Aj	Am	Aj	Am
2008/07											0	30				
2008/08											0	88				
2008/09											0	53				
2008/10											0	36				
2008/11											0	48				
2008/12											0	40				
2009/01											0	629	0	52		
2009/02			144	39			148	0	3	50	0	306				
2009/03	100	0	130	211			2	7	14	741	0	234				
2009/04			15	53					0	80	0	122				
2009/05							0	5	0	154	0	149				
2009/06			1	22			0	8	0	534	0	246				
2009/07			0	13			0	256	0	319	0	47				
2009/08			0	7			0	10	0	281	0	32				
2009/09			0	17			0	42	0	94	0	15	0	41		
2009/10			0	3			0	9	0	120			0	163		
2009/11			52	0			4	8					0	48		
2009/12			288	13			16	10	0	166			0	50		
2010/01			176	0			45	0	0	57			0	143		
2010/02			600	0			51	39	0	106						
2010/03	100	0	715	48			0	13	2	270						
2010/04									0	339			0	55		
2010/05																
2010/06																
2010/07					0	206			0	200						
2010/08					0	294			0	258						
2010/09					0	88			0	434						
2010/10					0	30			5	576						
2010/11					87	345			2	284						
2010/12					319	88			0	72						
2011/01	50	0			368	88			3	292						
2011/02	50	0			151	425			1	270						
2011/03	50	0			3	26			7	248						
2011/04	50	0			3	26			7	332						
2011/05	46	4			3	33			0	469					0	50
2011/06					1	33			0	203						
2011/07					0	138			0	177						
2011/08					2	21										
2011/09					1	78										
2011/10					3	9									0	100
2011/11					272	11									0	100
2011/12					112	15									0	100
Total	446	4	2121	426	1325	1954	266	407	44	7126	0	2075	0	552	0	350

SH: Shanghai; DS: Danshui R.; YL: Yilan R.; DG: Donggang R.; SL: Siouguluan R.; CY: Cagayan R.; BY: Buayan R.; MD: Manado; Aj: *A. japonica*; Am: *A. marmorata.*

**Figure 2:**
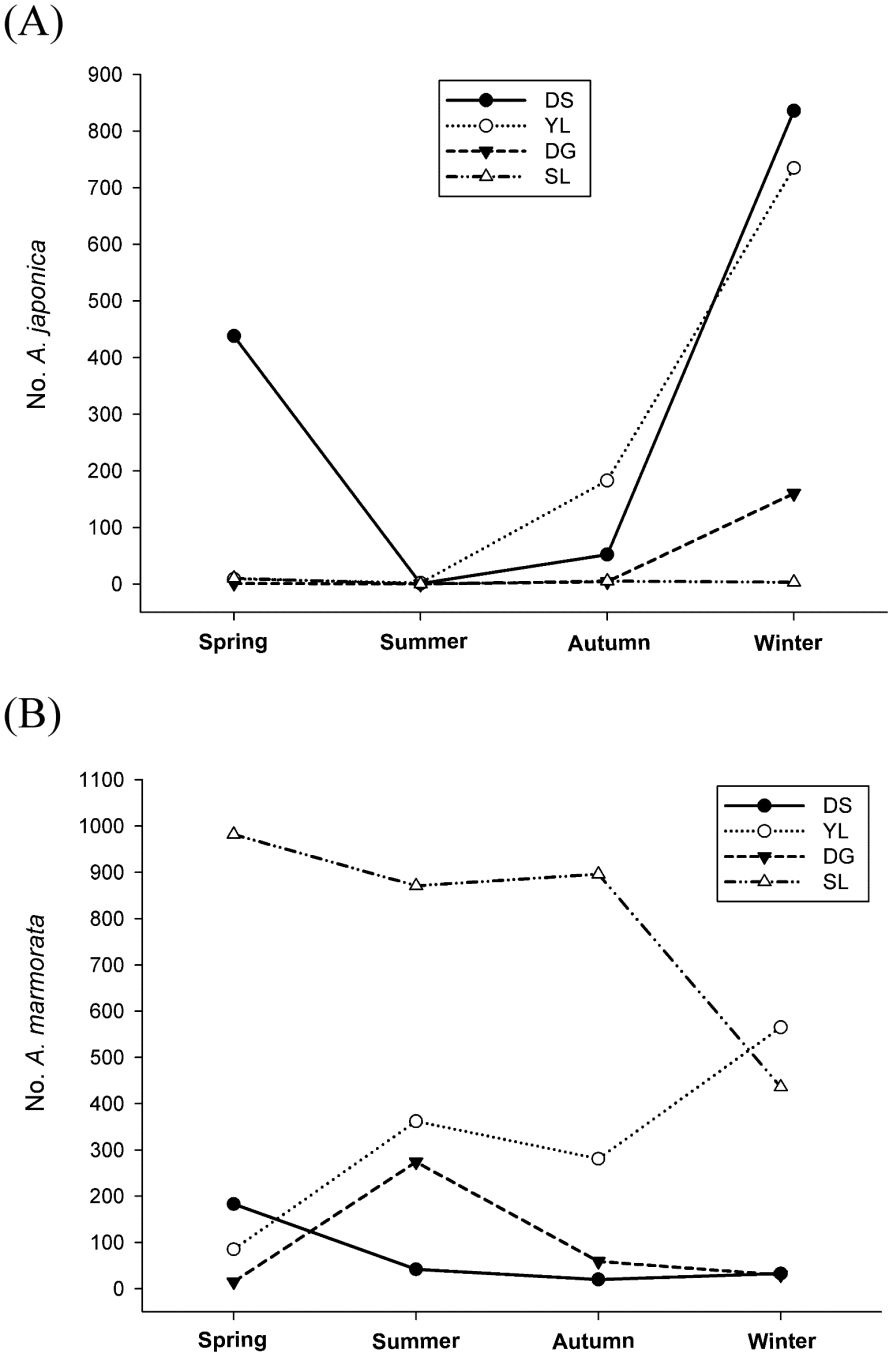
Catch numbers of *A. japonica* (A) and *A. marmorata* (B) glass eels in spring (March–May), summer (June–August), autumn (September–November), and winter (December–February). DS: Danshui R.; YL: Yilan R.; DG: Donggang R.; SL: Siouguluan R.

Kimura et al. [25] reported that eel larvae exhibit vertical migration behavior and may move to near-surface seawater (∼50–100 m) at night–the depth affected by northward Ekman transportation. The smaller larval size of *A. marmorata* compared with that of *A. japonica* might reduce their migration up to the surface Ekman layer at night. Thus, most *A. japonica* larvae are thought to enter the Kuroshio and reach their East Asian habitats. In contrast, *A. marmorata* larvae can enter both the northbound and southbound flows to reach their growth habitats [25]. On the other hand, Kuroki et al. [11] suggested that *A. marmorata* might tend to spawn further south in the NEC than *A. japonica*, thus enhancing their southward transport. This hypothesis could well explain the relative abundances of these 2 species in the northern and southern areas of East Asia. However, *A. marmorata* is also found to be abundant in Luzon Island, Philippines, where the Kuroshio passes by [26], whereas *A. japonica* is unusually rare throughout the Philippines and Indonesia. Some additional biotic/abiotic factors may also affect the distinct distribution patterns of these 2 species.

Previous studies have demonstrated that the feeding, swimming, pigmentation, and otolith growth of Japanese glass eels are hampered under low water temperatures [24], [27], [28]. The time lag in recruitment of Japanese glass eels to Northeast Asian habitats in winter can be accounted for by a longer leptocephalus stage combined with a low temperature-driven delay to upstream migration [24]. Therefore, it is possible that behaviors such as the temperature preference of the glass eels may help determine recruitment dynamics. In East Asia, the coastal waters of Taiwan in winter are affected by the cold northeastern monsoon and cold Chinese coastal water as well as the warm Kuroshio and its branch waters, thus forming a significant thermal front in the waters around Taiwan [5], [29], [30]. Furthermore, both *A. japonica* and *A. marmorata* are abundant in the rivers in Taiwan. The geographical location of Taiwan makes it an excellent site for investigating these environmental effects on the recruitment dynamics of these eel species. Thus, the present study aimed to clarify if the water temperature and oceanic current regime may affect the glass eel recruitment of *A. japonica* and *A. marmorata* by examining their temperature preferences and relative abundances in Taiwan, Philippines, China, and Indonesia as well as the available oceanic currents in East Asia.

**Table 3:** Survival rate (%) of *A. marmorata* at different water temperatures (5°C, 10°C, 15°C, or 20°C) and salinity (0%, 50%, or 100% seawater).

Temperature Salinity	5°C	10°C	15°C	20°C
100% seawater	0	16	45	100
50% seawater	0	100	100	100
0% seawater	0	100	100	100

Each group has 200 individuals (combining 2 experiments). Samples were kept in the dark without feeding for 1 week.

## Materials and Methods

### Sample Collection

Glass eels from Taiwan were collected monthly from the estuaries of the Danshui River (Northwestern Taiwan), Donggang River (Southwestern Taiwan), Yilan River (Northeastern Taiwan), and Siouguluan River (Eastern Taiwan) (Fig. 1). Glass eels were caught using fyke nets at night between February 2009 and December 2011. One to three samplings taking 2 hours each were performed every month at each location. The data for each monthly collection were averaged. By using fyke nets, glass eels from the Philippines were collected from the estuary of the Cagayan River in northern Luzon Island and from the Buayan River in Mindanao Island (Fig. 1). Between July 2008 and April 2010, glass eels were purchased every month from the local fishermen. Glass eels were also collected from Manado in Indonesia (by using hand nets) and Shanghai in China (by using fyke nets) (Fig. 1). After collection, the glass eels were immediately preserved in 95% ethanol. Glass eel collections are allowed in these countries without need of any permission.

**Figure 3:**
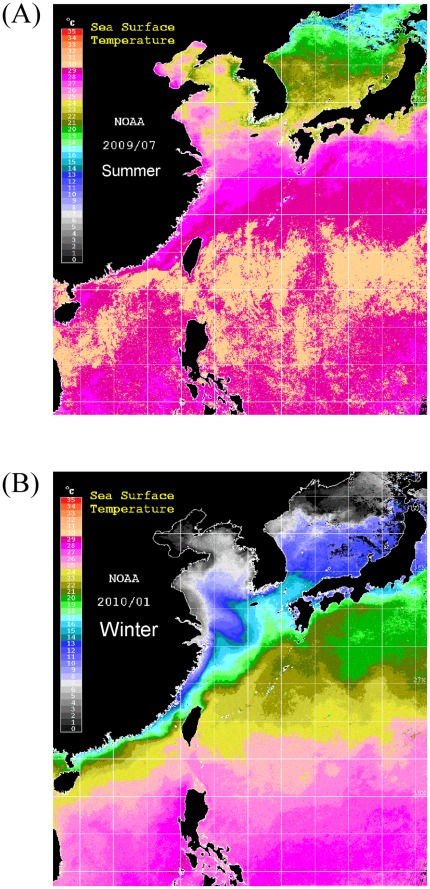
Map showing the mean SST images of the East Asian continental shelf in summer (July 2009) (A) and winter (January 2010) (B). The image was obtained using the National Oceanic and Atmospheric Administration AVHRR sensor data.

### Species Identification

Eel species were identified using both morphological and genetic methods. The total length, pre-dorsal length, and pre-anal length (to the nearest 0.1 mm) of each glass eel were measured under a stereomicroscope. The following equation was used to calculate the fin difference as an index to classify whether an eel was long finned or short finned: AD/TL × 100%  =  fin difference, where AD is the vertical distance from the origin of the dorsal fin to the anus and TL is the total length. Moreover, morphological characteristics were studied by determining the presence or absence of caudal cutaneous pigmentation according to Tabeta et al. [26]. *A. bicolor pacifica* (short finned) and *A. japonica* (no pigmentation on tail) are easily distinguished from other species. To identify other possible eel species in Taiwan, China, and Luzon Island, one piece of muscle from each individual was removed and used for genetic identification. Primer sets for cytochrome *b* (universal forward primer + species-specific reverse primer) were designed from *A. marmorata*, *A. celebesensis*, and *A. luzonensis* (synonyms of *A. huangi*) (Table 1); *A. luzonensis* is a newly reported eel species found around Luzon Island and Taiwan [31], [32]. All glass eel specimens caught in Mindanao Island and Indonesia were PCR amplified using a universal cytochrome *b* primer set (Table 1) followed by gene sequencing for direct species identification. A commercial DNA purification and extraction kit (Bioman Scientific Ltd., Taiwan) was used for genomic DNA extraction. PCR was performed as described previously [33]. The PCR products were then subjected to 2% agarose gel electrophoresis for band checking. Samples that failed to show species-specific bands were then PCR amplified using a universal primer set of cytochrome *b* (Table 1) for direct species identification.

**Figure 4:**
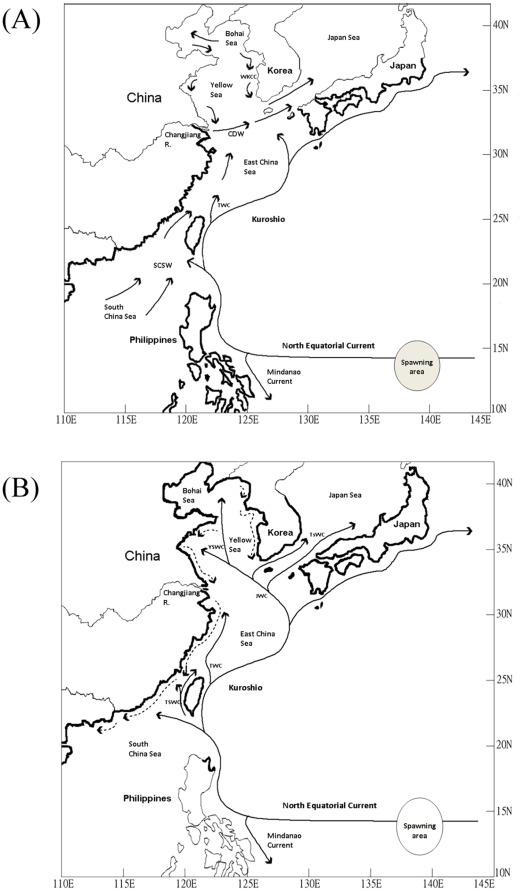
Maps showing the oceanic currents and geographical distributions of *A. japonica* and *A. marmorata* in East Asia. Oceanic and coastal currents in summer (June–August), and the distribution of *A. marmorata* (bold lines) in East Asia were shown in (A). Oceanic and coastal currents in winter (December–February), and the distribution of *A. japonica* (bold lines) in East Asia were shown in (B). CDW: Changjiang Diluted Water; SCSW: South China Sea Water; TSWC: Taiwan Strait Warm Current; TWC: Taiwan Warm Current; JWC: Jeju Warm Current; TsWC: Tsushima Warm Current; YSWC: Yellow Sea Warm Current; WKCC: Western Korea Cold Current.

### Temperature and Salinity Experiments with A. marmorata

By using fyke nets, *A. marmorata* glass eels were collected from the coastal waters of Yilan, Taiwan in August 2010. The glass eels were kept in a bag with saline and oxygen, and were immediately transported to the laboratory at the Institute of Fisheries Science, National Taiwan University, Taipei, Taiwan. Before the experiments, the glass eels were divided and kept in 12 aqua tanks in the dark without feeding. Each tank contained 100 individuals. They were gradually acclimatized from room temperature to 5°C, 10°C, 15°C, and 20°C for 1 day under varying salinity at 0, 17, or 35 psu for each temperature. After 1 week, the survival rates with respect to salinity and temperature for each tank were determined. Differences in the survival rates among temperature and salinity groups were tested by two-way analysis of variance (ANOVA). Data were considered significant at *p*<0.05. The same experiment was repeated in November 2011. The experiments were performed in fish rearing laboratory of Institute of Fisheries Science, National Taiwan University following the protocol for the care and use of laboratory animals of the National Taiwan University. The animal use has been reviewed and approved by the Institutional Animal Care and Use Committee (IACUC Number: 98-126, by chairman Jih-Tay Hsu on March 2010). Every effort was made to minimize suffering.

### Current and Temperature Patterns of East Asian Waters

The average sea surface temperatures (SSTs) in East Asia in July 2009 (summer) and January 2010 (winter) were estimated from the advanced very-high resolution radiometer (AVHRR) sensors on the TIROS-N series satellite of the National Oceanic and Atmospheric Administration (NOAA) by the Department of Environmental Biology and Fisheries Science, National Taiwan Ocean University, which provides high-quality SST data with a spatial resolution of 1.1 km. The major surface currents of the East Asian continental shelf in winter (December–February) and summer (June–August) were modified based on Chen’s studies [29], [34].

**Figure 5:**
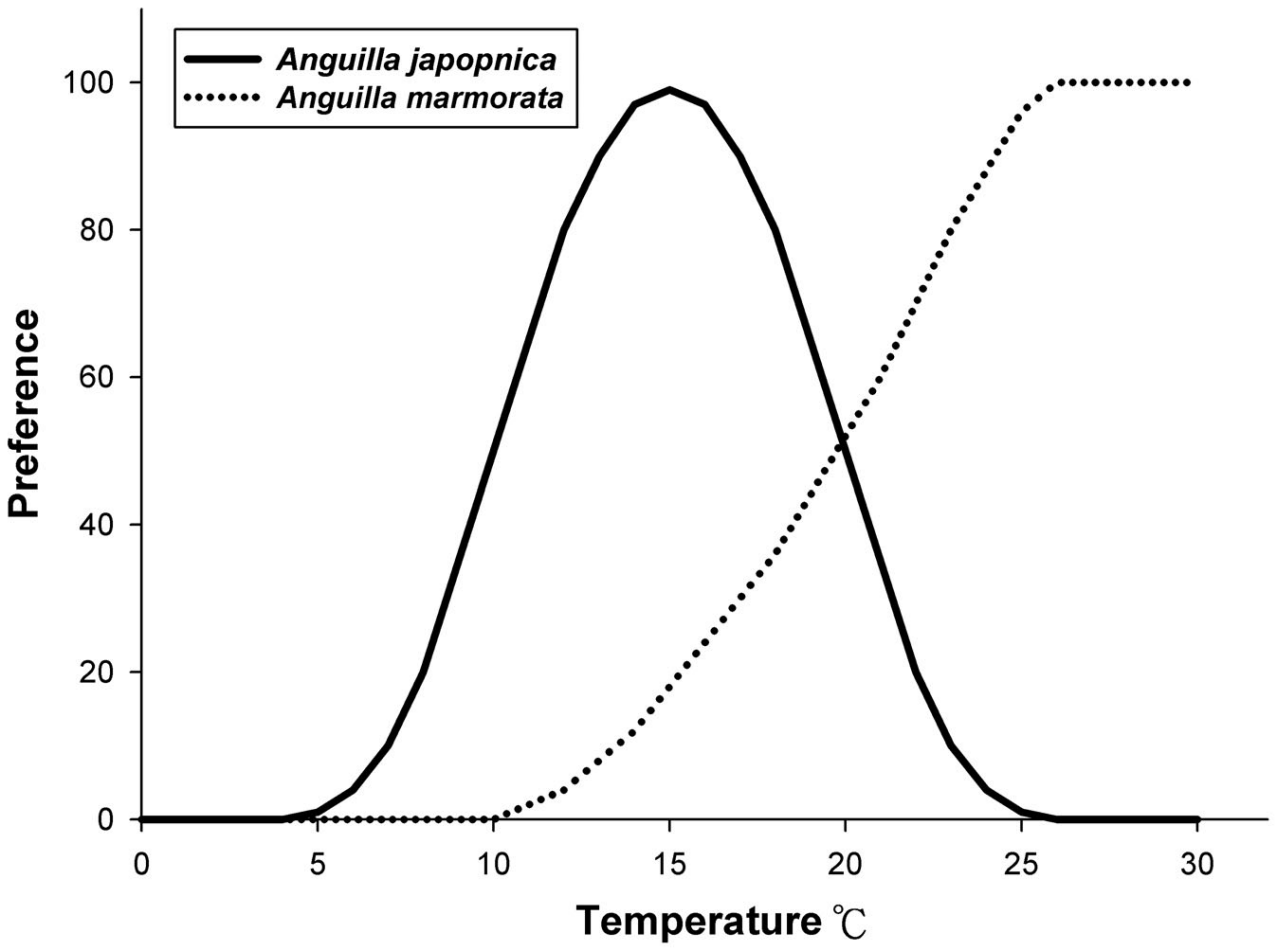
Water temperature preferences for *A. japonica* and *A. marmorata* recruitments.

## Results

### Monthly Glass Eel Composition by Location

Glass eel sampling and monthly composition by location are shown in Table 2 and Fig. 2. A total of 4 *Anguilla* species were identified in Taiwan: *A. japonica*, *A. marmorata*, *A. bicolor pacifica*, and *A. luzonensis*. Three eel species were found in Luzon Island: *A. marmorata*, *A. bicolor pacifica*, and *A. luzonensis*. Five eel species were identified in Mindanao Island: *A. marmorata*, *A. bicolor pacifica*, *A. luzonensis*, *A. celebesensis*, and *A. interioris*. Four species were found in Manado, Sulawesi, Indonesia: *A. marmorata*, *A. bicolor pacifica*, *A. celebesensis*, and *A. interioris*. Only *A. japonica* and *A. marmorata* were identified in Shanghai, China. In this study, all eel species other than *A. japonica* and *A. marmorata* were excluded from analysis.

The Siouguluan River, Taiwan was dominated by *A. marmorata* (>99%) throughout the year; only few *A. japonica* glass eels were found in winter (Table 2; Fig. 2). In the Yilan River, Taiwan, *A. marmorata* was found throughout the year, whereas *A. japonica* was abundant in winter (Table 2; Fig. 2). In the Donggang River, Taiwan, *A. marmorata* was observed during most of the year and was more abundant in summer, whereas *A. japonica* was more abundant in winter (Table 2; Fig. 2). In the Danshui River, Taiwan, *A. japonica* was far more abundant than *A. marmorata* in winter, and *A. marmorata* was found almost year round but was more abundant in spring (Table 2; Fig. 2). In Shanghai, China, *A. marmorata* was only observed in May during the sampling period from January to May (Table 2). In the Philippines and Manado, *A. marmorata* was the most dominant eel species and no *A. japonica* specimens were found (Table 2).

### Temperature and Salinity Tolerance of *A. marmorata*

*A. marmorata* glass eels were maintained at 5°C, 10°C, 15°C, or 20°C; each temperature group was kept under 3 salinity conditions: 0% (0 psu), 50% (17 psu), or 100% (35 psu) seawater. The same experiment was repeated on November 2011, and similar results were obtained. Thus, the data were pooled for analysis. All glass eels died within 1 day at 5°C in all salinity groups (Table 3). In the 10°C and 15°C groups, glass eels only died in 100% (35 psu) seawater (Table 3). In the 20°C groups, no glass eels died at any salinity level after 1 week of observation (Table 3). The effect of water temperature on the survival rate of glass eels was significant (*p = *0.01, F = 9.88, df = 3). Although the effect of salinity on the survival rate of glass eels was not significant overall (*p = *0.14, F = 2.76, df = 2), *A. marmorata* glass eels exhibited temperature-dependent mortality in 100% seawater (Table 3).

### SST and Surface Currents in East Asia

The mean SST of East Asian coastal waters in summer (July 2009) and winter (January 2010) are shown in Fig. 3. During summer, the mean SST was >24°C in all areas except the Japan Sea (Fig. 3A). However, in winter, when the warm Kuroshio waters flow northward and the cold coastal waters flow southward, clear temperature fronts exist on the continental shelf of East Asia. The SST around northern Luzon Island was high (>25°C) in winter but <20°C on the coast of Southern China and Pacific coast of Japan and <15°C on the southern coast of Korea and central coast of China. SST was usually <5°C north of the Changjiang River in China and on the western coast of Korea (Fig. 3B). In the coastal waters around Taiwan, the mean SST in winter was higher in southeast sites (>24°C) than in northwest sites (<20°C), forming a clear temperature front (Fig. 3B).

In summer (June–August), the southwest warm monsoon prevails in East Asia. The main stream of the Kuroshio in the East China Sea typically flows along the steep continental slope [29]. The Taiwan Strait waters are dominated by waters from the South China Sea (Fig. 4A). The South China Sea Water flows northward from the South China Sea, passes through the Taiwan Strait, and finally merges with the Changjiang Diluted Water (CDW) (Fig. 4A). The Taiwan Warm Current flows northward and merges together with waters from the Taiwan Strait. Because of the high discharge of the outflow from the Changjiang River, it forms the CDW and flows eastward toward Jeju Island and the Japan Sea (Fig. 4A).

In winter (December–February) in East Asia, the Kuroshio intrudes into the Luzon Strait, which flows westward toward southern China and northward along the Taiwan Strait, forming the Taiwan Strait Warm Current (Fig. 4B). The Taiwan Warm Current flows northward toward the Changjiang River estuary throughout the year, even during strong northeast cold monsoons in winter [35]. The Kuroshio branch water turns westward at Kyushu Island, Japan, and forms the Jeju Warm Current (Fig. 4B). The Jeju Warm Current bifurcates into the eastward Tsushima Warm Current and the westward Yellow Sea Warm Current [36], [37]. Part of the Yellow Sea Warm Current flows into the Bohai Sea to compensate for the southward flow of cold coastal waters (Fig. 4B) [38]. The strong northeastern winds in winter push the coastal cold waters flowing southward along the coasts of China; they also push the eastern branch of the coastal waters of the Bohai Sea, namely, the West Korea Coast Current, to follow the western coast of Korea southward.

## Discussion

*A. marmorata* and *A. japonica* have overlapping spawning sites and comparable larval durations [3], [12], [22]. In theory, glass eels of these species should be transported by the Kuroshio and its branch waters to similar habitats in East Asia. However, the present results show that their distribution patterns on the Kuroshio route are quite different. *A. marmorata* was found in abundance throughout the year in Luzon Island and the Siouguluan River, Taiwan, whereas *A. japonica* was rare in these locations [26] (Table 2). In the Donggang River in Taiwan and Yakushima Island in Southern Japan, *A. marmorata* and *A. japonica* were mainly found during summer and winter, respectively [39] (Table 2). In the Danshui River, Taiwan, *A. marmorata* recruitment occurred mainly in spring, while *A. japonica* recruitment occurred in winter and spring. In the Yilan River, Taiwan, *A. marmorata* was abundant almost throughout the year, while *A. japonica* was abundant in winter. It is known that the time differences among the recruitment sites for glass eels in Taiwan are only 1–3 weeks [5]. However, the distribution patterns and timings of both eel species in Taiwan are dramatically different. Furthermore, in Shanghai, only *A. japonica* was observed in winter and *A. marmorata* was found in May (spring). *A. japonica* glass eels were not found in the Philippines or Indonesia (Fig. 1, Table 2). These phenomena strongly suggest that some biotic or environmental factors may be involved in the distinct distribution patterns of these species in East Asia.

The salinity and temperature tests in the present study clearly show that *A. marmorata* cannot survive in cold waters regardless of salinity; the species also exhibits temperature-dependent mortality in seawater. In contrast, a previous study indicates that *A. japonica* glass eels can endure temperatures of 4°C for several months [24]. Meanwhile, the mean SST in Southeastern Taiwan, the Philippines, and Indonesia are all well above 24°C in winter (Fig. 3B)–locations where the *A. marmorata* glass eels but not the *A. japonica* glass eels are predominant. In contrast, the mean SSTs in northwestern Taiwan, China, Japan, and Korea are all well below 20°C in winter (Fig. 3B) where the *A. japonica* glass eels but not the *A. marmorata* glass eels are predominant. Therefore, the 24°C SST isotherm clearly separates the recruitment of these 2 eel species. The failed acclimation in cold seawater and predominant recruitment in hot areas indicate that *A. marmorata* glass eels prefer higher temperatures for recruitment. In contrast, the good acclimation of *A. japonica* glass eels in cold seawater and failed recruitment in hot areas indicate that *A. japonica* glass eels prefer lower recruitment temperatures. In Yakushima Island, *A. marmorata* glass eels are mainly found during hot summers [39]. Recruitment is concentrated during the hot season at higher latitudes. The lowest mean SST in the Danshui River, Taiwan, in winter coincides with the relative abundance of *A. japonica* glass eels over those of *A. marmorata*. On the other hand, the mean SST in the Yilan River in winter is at least 2°C lower than that in the Siouguluan River due to the upwelling effect of the Kuroshio there (Fig. 3B). The contrast recruitment patterns of both glass eels between the nearby Yilan and Siouguluan Rivers in Taiwan highlights the significant effect of seawater temperature on glass eel recruitment dynamics.

*A. japonica* glass eels spawn mainly in summer and are distributed in Taiwan, China, Korea, and Japan [40], [41]. Their recruitment occurs first in Taiwan around late October and ends in Northern China and the western coast of Korea around May [24]. The distribution of *A. japonica* glass eels matches the recorded flow of oceanic currents, indicating the strong dependence of larval transportation on available oceanic currents (Fig. 4B) [5]. Sinclair [42] proposed the “member–vagrant” hypothesis, which states that the marine larvae that survive to settle in appropriate habitats are retained by indicated oceanic currents; in this case, *A. japonica* glass eels transported to the appropriate habitats are “members,” while those transported to unsuitable habitats are “vagrants.” The recruitment of *A. japonica* glass eels preferably occurs in estuaries with mean SSTs <24°C (Fig. 5); those transported by the Mindanao Current and Kuroshio to non-preferable areas may not actively perform upstream migration in estuaries and are thus retained at sea. Although yellow-stage eels are able to survive in brackish water and seawater [43]–[45], seawater seems to be an unsuitable niche for marine anguillid eels since very few adults can be caught in the seawaters of tropical areas.

Glass eels could sense changes in water temperature as little as 1°C [46]. Matsui [47] found that Japanese glass eels can be caught in rivers with temperatures above 8–10°C. Han [24] suggested that Japanese glass eels do not migrate upstream when temperatures are below 5°C. In this study, the recruitment of Japanese glass eels ceased when the temperature exceeded 24°C. Based on Xiong et al. [48] and interviews with fishermen, the most suitable recruitment temperature for Japanese glass eels is between 14°C and 18°C. Thus, the recruitment tendency of Japanese glass eels is dependent upon temperature, forming a bell-shaped curve (Fig. 5). This explains why Japanese glass eels are very rare around Luzon Island and why there is almost no recruitment in Indonesia. Similar bell-shaped recruitment temperature preferences also exist in 2 other temperate eels, *A. australis* and *A. dieffenbachii*, which have optimal recruitment temperatures around 16.5°C and inhibitory migration temperatures of <12°C and >22°C [49]. When cold fronts invade Taiwan, the catch of *A. marmorata* glass eels decreases significantly; meanwhile, glass eels are far more abundant in Eastern Taiwan (i.e., the Siouguluan and Yilan Rivers) than in Northwestern Taiwan (Danshui River). In Shanghai, no *A. marmorata* glass eels were caught during the winter, suggesting that its recruitment ceases at temperatures below 15°C. Taken together, the higher the water temperature, the more upstream activity *A. marmorata* glass eels perform (Fig. 5). The opposite water temperature preferences of the *A. marmorata* and the *A. japonica* glass eels is possibly one important behavior trait that shapes their distinct dispersal patterns in East Asia.

The mean SSTs in Korea and the coasts north of the Changjiang River in China are <5°C in winter–a temperature that does not allow the survival of *A. marmorata* glass eels (Fig. 3B); the mean SSTs around Japan, the central areas of China, and the East China Sea are generally <15°C, which is also a temperature unsuitable for their survival. Thus, in winter, *A. marmorata* glass eels are “screened out” in large numbers in the northern areas of East Asia even if they are transported to these areas. However, in summer, the SSTs in all of East Asia are suitable for *A. marmorata* glass eel recruitment. Despite this, *A. marmorata* glass eels are seldom found in China or Korea during this time. In summer, there is a strong coastal current coming from the South China Sea that flows northward along the Chinese coast to the Changjiang River where it finally merges with the CDW (Fig. 4A). The outflow of the Changjiang River flows northeastward toward Jeju Island; these 2 waters efficiently block the invasions of Kuroshio and its branch waters into China and Korea (Fig. 4A). Thus, the approach of *A. marmorata* glass eels to China and Korea coasts is impaired in summer. Indeed, in Taiwan, *A. marmorata* glass eels are far more abundant in eastern sites than in western ones in summer (Fig. 2B; Han, per. comm.); this suggests that the Taiwan Strait Warm Current, which delivers glass eels to the Taiwan Strait [5], is also blocked by the South China Sea Water. Taken together, cold SSTs inhibit the distribution of *A. marmorata* glass eels to Northern China and Korea in winter. Furthermore, in summer, the coastal current from the South China Sea and CDW also block their approach to China and Korea. Thus, *A. marmorata* glass eels are mainly distributed in areas along the main stream of the Kuroshio and Mindanao Current (Fig. 1, 4A).

Some anguillid species may also share overlapping spawning areas and be transported by similar oceanic currents. For example, the American eel *A. rostrata* and the European eel *A. anguilla* both spawn in the Sargasso Sea [50]. The significantly longer larval duration of the European eel compared to that in the American eel allows their offspring to disperse separately on either side of the Atlantic Ocean [51]. Similarly, both *A. reinhardtii* and *A. australis* are dispersed by the South Equatorial Current [2]; the former ranges mainly between 20° and 34° S (tropical/subtropical waters) but the latter is distributed mainly from 35–44° S (temperate waters) in Australia [52], [53]. The longer duration of marine larval period in *A. australis* compared to that in *A. reinhardtii* is thought to be responsible for determining its geographical distribution [16]. On the other hand, the *A. bicolor bicolor* and *A. mossambica* glass eels supposedly spawn east of Madagascar [54]; the former species reaches the rivers of the eastern coast of Africa preferentially north of 20° S, while those of the latter are south of 20° S and are predominant in South Africa rivers [55], [56]. However, both species have comparable larval durations [57]. The distinct geographical distributions of these 2 eel species may possibly be because of their different temperature preferences, similar to the case in the present study.

In conclusion, the clear thermal fronts of seawaters around Taiwan in winter are important for the distinct biogeography of *A. marmorata* and *A. japonica*. Biological factors such as the length of larval duration, behavioral traits of the leptocephali, and temperature preferences of the glass eels combined with environmental factors such as spawning locations and available oceanic currents act together as limiting factors to shape the realized geographic distributions of these species. This provides some clues for other marine fish species that share overlapping spawning areas but are recruited to different habitats.
